# EGFR of platelet regulates macrophage activation and bacterial phagocytosis function

**DOI:** 10.1186/s12950-024-00382-1

**Published:** 2024-04-17

**Authors:** Shuhua Luo, Riping Xu, Pengyun Xie, Xiaolei Liu, Chunxiu Ling, Yusha Liu, Xuedi Zhang, Zhengyuan Xia, Zhanghui Chen, Jing Tang

**Affiliations:** 1 Department of Anesthesiology, Affiliated Hospital of Guangdong Medical University, 524000 Zhanjiang, Guangdong China; 2 Guang Dong Medical University, 524000 Zhanjiang, Guangdong China; 3 Zhanjiang Institute of Clinical Medicine, Zhanjiang Central Hospital, Guangdong Medical University, 524000 Zhanjiang, Guangdong, China

**Keywords:** Platelet, EGFR, Macrophage, Phagocytosis, Pyroptosis, Sepsis

## Abstract

**Background:**

Beyond their crucial role in hemostasis, platelets possess the ability to regulate inflammation and combat infections through various mechanisms. Stringent control of macrophage activation is essential during innate immune responses in sepsis. Macrophages are considered crucial phagocytic cells that aid in the elimination of pathogens. Platelet interactions with monocytes-macrophages are known to be significant in the response against bacterial infections, but the primary mediator driving these interactions remains unclear. EGFR plays critical role in the regulation of inflammation and infection through various mechanisms.

**Results:**

The overexpression of platelets by thrombopoietin (TPO) leads to the sequestration of both pro-inflammatory (IL-6/IL-1) and anti-inflammatory (IL-10) cytokines in the organ tissue of septic mice. Epidermal growth factor receptor (EGFR) is critical for platelet activation in sepsis. EGFR-licensed platelets enhance macrophage immune function, including the production of reactive oxygen species (ROS) and the clearance of bacteria. Platelet EGFR also induces M1 macrophage polarization by increasing the expression of inducible nitric oxide synthase (iNOS) and CD64.

**Conclusion:**

EGFR can activate platelet immune function. Moreover, activated platelets efficiently regulate bacterial phagocytosis and pro-inflammatory function of macrophages through an EGFR-dependent pathway.

**Supplementary Information:**

The online version contains supplementary material available at 10.1186/s12950-024-00382-1.

## Introduction

Platelets circulate in the vessel with the ability to bind to damaged vessels and preventing bleeding rapidly [1]. Beyond their crucial role in hemostasis, platelets possess the ability to regulate inflammation and combat infections through various mechanisms [1–3]. Platelets can recognize pathogens in blood and triggering various mechanisms that bolster innate resistance to infection [2, 4, 5]. Platelets serve as immune-sensing cells with prominent expression of inflammatory related molecules, such as Toll-like receptors (TLRs), adhesion molecules, and signaling mediators crucial for the recruitment of neutrophils and monocytes [4, 6, 7].

Macrophages are regarded as vital phagocytic cells which help to eliminate pathogens. In response to prevailing stimuli within inflammatory microenvironments, macrophages can undergo differentiation from circulating monocytes and become polarized toward either pro-inflammatory M1 or anti-inflammatory M2 phenotypes, respectively [8–10].

While platelet interactions with monocytes-macrophages are known to be significant in the response against bacterial infections, the primary mediator driving these interactions remains unclear [5, 11, 12]. Previous studies have provided limited and conflicting results, demonstrating both inhibition and induction of inflammatory cytokines when platelets are co-cultured with macrophages upon stimulation with lipopolysaccharide (LPS) [13, 14].

Numerous studies have indicated that platelets play a vital role in bacterial clearance and the resolution of inflammation by modulating macrophage responses. Platelets can suppress the release of pro-inflammatory molecules derived from macrophages, leading to the rescue of mice of septic shock from mortality [13]. These findings underscore the potentially beneficial therapeutic role of platelets in the management of severe sepsis in patients [13]. Circulating platelet can aggregate monocyte and exacerbate the inflammatory response in older patients with sepsis. Whereas this platelet-monocyte complex may serve as a prognostic indicator for septic mortality, the precise mechanisms underlying this phenomenon remain to be fully elucidated [15].

Epidermal growth factor receptor (EGFR) belongs to the ERBB family of tyrosine kinase receptors [16]. The EGFR signaling cascade serves as a pivotal regulator in various cellular processes, including proliferation, differentiation, division, survival, and the development of cancer [17, 18]. In addition to their significant role in cancer development, EGFR are also involved in the regulation of inflammation and infection through various mechanisms [19, 20]. Previous studies have demonstrated that phosphorylation of EGFR is necessary for TLR4-mediated activation of macrophages during sepsis [21]. In sepsis, EGFR can induce the apoptosis of CD4 + T lymphocytes by promoting the Warburg effect through TBK1/Glut1 signaling pathway [22]. Nevertheless, the regulatory role of EGFR on platelets in the context of sepsis remains largely unexplored.

We propose that EGFR might play a crucial role in platelets to modulate the macrophage program towards a pro-inflammatory M1 phenotype during sepsis. This modulation could be essential in limiting bacterial dissemination, thereby serving as a critical mechanism in the host response to sepsis. To gain deeper insights into the role of EGFR in platelets during sepsis, we treated platelet with EGFR inhibitors treatment. Subsequently, we assessed the change in platelet co-incubated macrophage immune function, including polarization, bacterial clearance, and death manners. This study offers novel insights into platelet biology, emphasizing the significance of EGFR in facilitating platelet activation and modulating macrophage immune function during sepsis.

## Results

### TPO-elevated platelets improve the survival rate and alleviates organ injury in CLP sepsis mice

**Fig. 1:**
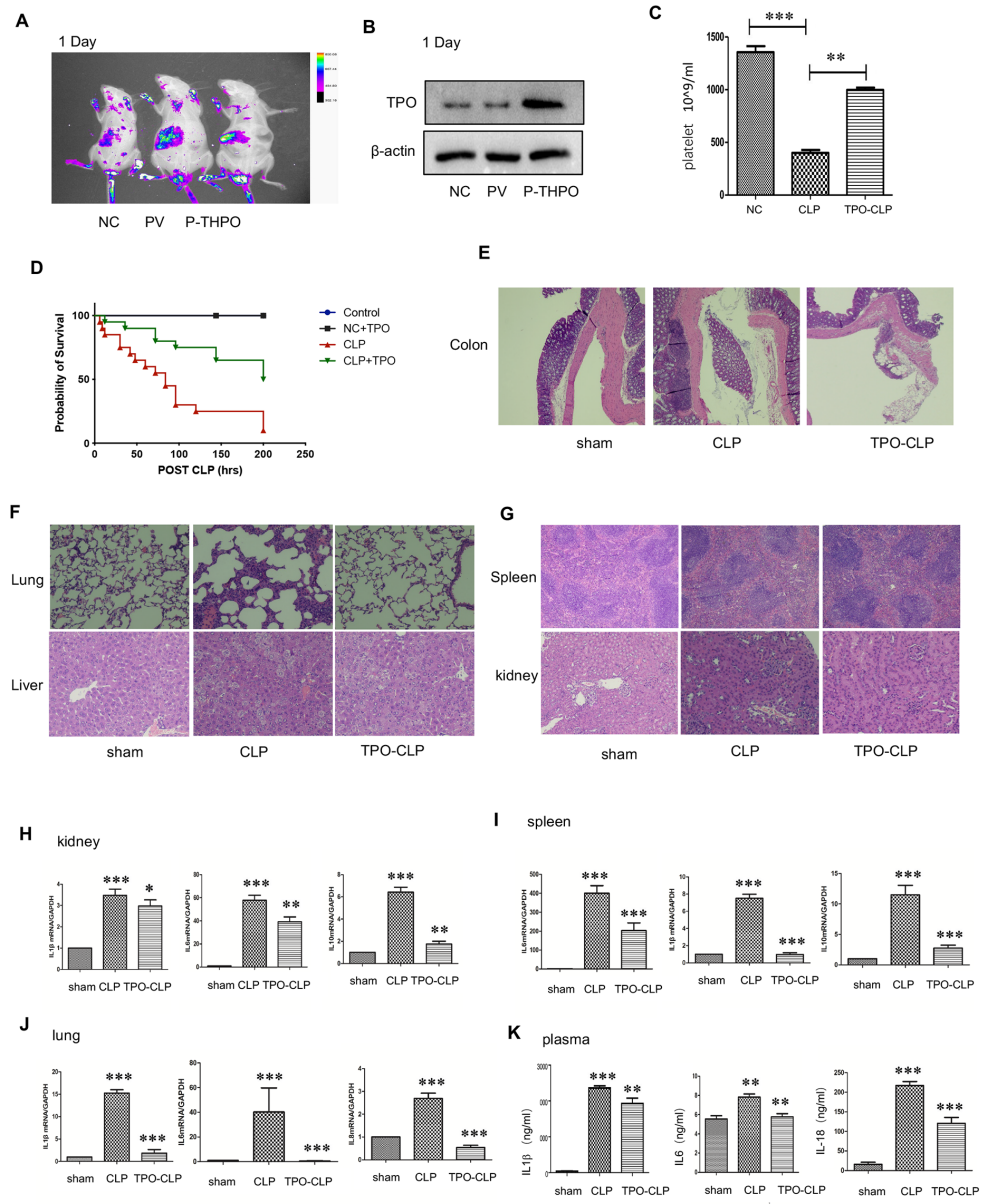
TPO-elevated platelets improve the survival rate and alleviates organ injury in sepsis. (**A**) The distribution of GFP green fluorescence in mouse liver was detected by small animal imaging system 5 days after TPO- overexpression plasmid injection;. (**B**) The expression level of hepatic thrombopoietin (TPO) in mice was confirmed by Western blot 5 days after TPO-overexpression plasmid injection;. (**C**) platelet counts in peripheral blood of mice 5 days after TPO-plasmid injection;. (**D**) Survival of septic mice (*n* = 20 per group) after CLP challenge;. (**E-G**) H-E staining of mouse colon, lung, liver, spleen and kidney sections 24 h after CLP;. (**H-J**) mRNA expression of inflammatory factors in mouse kidney, spleen and lung tissue 24 h after CLP;. (**K**) ELISA results of mouse plasma inflammatory factors IL1β, IL6 and IL18 24 h after CLP. (C: two tailed t-test; J-H, M: one-way ANOVA analysis. * represents *P* < 0.05, ** represents *P* < 0.01, and *** represents *P* < 0.001)

The impact of platelets in sepsis remains a topic of debate and controversy [2, 13]. To assess the in vivo immune function of platelets under septic conditions, we developed platelet overexpression mice by administering thrombopoietin (TPO) overexpression lentiviral plasmid via tail vein injection (Fig. 1A, Fig.S1A-C). We verified that TPO expression in hepatocytes was elevated compared to the untreated group (NC) and sham group (PV) 5 days after the tail vein injection (Fig. 1B).

Concurrently, the total number of platelets in the peripheral blood of mice was increased compared to WT controls (Fig. S1D-E). We observed a significant decrease in platelet levels in CLP septic mice, while TPO overexpression effectively increased platelet numbers in CLP sepsis (Fig. 1C). Remarkably, platelet overexpression induced by TPO administration increased the survival of CLP mice (Fig. 1D).

Acute organ dysfunction is a critical manifestation of severe sepsis, and the rapid elevation of inflammatory cytokines often exacerbates organ injury [23]. Next, we analyzed organ injury in septic conditions and found that elevated platelet level induced by TPO administration could mitigate tissue damage in the lung, kidney, liver, and spleen of CLP mice (Fig. 1E-G). In septic conditions, platelets elevated by TPO administration markedly diminish both pro-inflammatory and anti-inflammatory responses in these organs (Fig. 1H-J). Additionally, platelets elevated by TPO reduced the levels of IL-1β, IL-6, and IL-18 cytokines in plasma, as determined by ELISA in CLP mice (Fig. 1K). These data suggest that platelets likely play a protective role in sepsis.

### EGFR regulates activation of platelets in sepsis

**Fig. 2:**
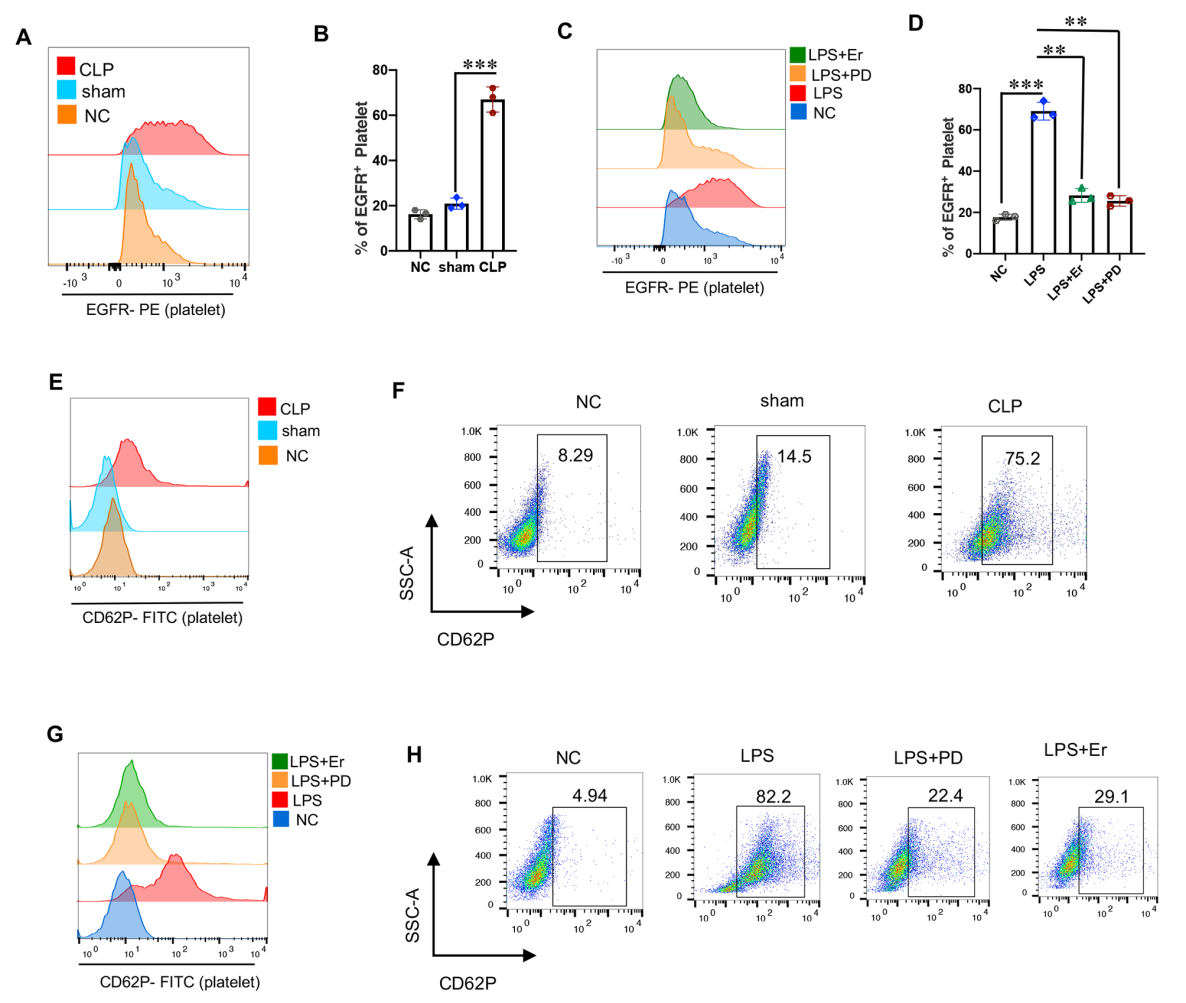
EGFR regulates activation of platelets in sepsis. (**A-B**) Membrane EGFR expression of platelets in sepsis in vivo;. (**C-D**) Membrane EGFR expression of isolated platelets treated with LPS in vitro;. (**E-F**) Membrane CD62P expression of platelets in sepsis in vivo;. (**G-H**) Membrane CD62P expression of isolated platelets treated with LPS in vitro. (B, D: two tailed t-test * represents *P* < 0.05, ** represents *P* < 0.01, *** represents *P* < 0.001.)

Platelets express many immune components and serve as immune-sensing cells. Specifically, toll-like receptors 4 and 2, complement receptors and adhesion molecules are reported to play important roles in platelets’ immune function [6, 7, 24]. Using flow cytometry, we confirmed the expression of EGFR in platelets, and notable increase in EGFR expression was observed in platelets from the CLP sepsis model (Fig. 2A-B). In vitro experiments further demonstrated that EGFR expression was heightened in isolated platelets from WT mice following LPS stimulation. This increase in EGFR expression could be inhibited by EGFR-specific antagonists PD-168,393 (PD) and Erlotinib (Fig. 2C-D). Surface expression of CD62P is indicative for platelet activation and positive mediator for interactions between platelets and leukocytes. FACS analysis revealed a significant increase in CD62P expression on platelets during sepsis (Fig. 2E-F). As anticipated, in vitro LPS treatment led to an increase in CD62P expression on platelets. Interestingly, treatment with PD or Erlotinib effectively inhibited the LPS-mediated activation of platelets (Fig. 2G-H). These findings demonstrate that EGFR could serve as a pro-activation receptor in platelets during sepsis.

### Platelet mediated bacteria phagocytosis of macrophage is dependent on EGFR

**Fig. 3:**
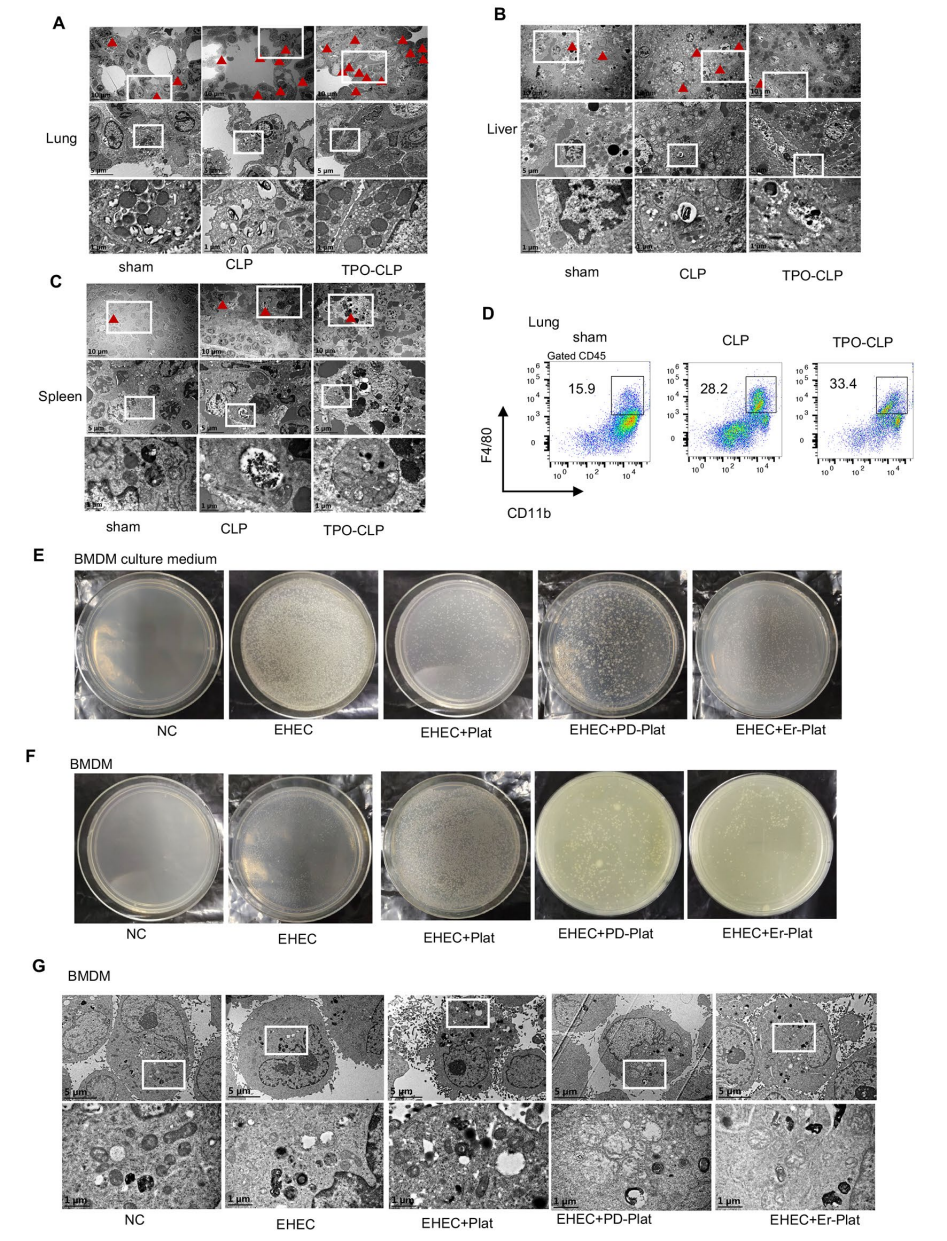
Platelet mediated bacteria phagocytosis of macrophage is dependent on EGFR. (**A-C**) Electron microscope image of mouse lungs, liver and spleen 24 h after CLP, red triangles mark macrophages;. (**D**) Macrophage population of lung tissue in CLP mice analyzed by flow cytometry;. (**E**) Bacterial plating of supernatants after 16 h of EHEC treatment of BMDM co-cultured with indicated platelets;. (**F**) BMDMs co-cultured with indicated platelets were treated with EHEC for 16 h, then washed with gentamicin for 1 h, and the cells were permeabilized for bacterial plating;. (**G**) Electron microscope image of BMDM treated with EHEC and platelets

Efficient phagocytosis of bacteria by macrophages is one of the most crucial mechanisms for host defense against pathogens in sepsis [9, 25]. Active interactions between platelets and macrophages are considered to play a significant role in the defense against bacterial infections [14, 26]. To investigate these possibilities, we initially conducted tissue electron microscopy analysis. Our findings revealed that macrophages exhibited enhanced activity in bacterial phagocytosis within the lung, liver, and spleen of platelet overexpression CLP mice compared to WT CLP mice. This enhanced activity was characterized by increased presence of granules, lysosomes, and digestive vesicles (Fig. 3A-C).

Furthermore, we observed that TPO-elevated platelets also recruited a greater population of macrophages in CLP mice (Fig. 3D). Platelets have been shown to express certain mediators crucial for recruiting neutrophils and monocytes [6], yet the underlying mechanism remains poorly understood. Given that EGFR can enhance platelet activation, we investigated the role of platelet EGFR in modulating macrophage immune function. We pre-treated isolated platelets with EGFR inhibitors PD-168,393 or Erlotinib for 1 h before co-incubation with bone marrow-derived macrophages (BMDMs), followed by stimulation with E. coli O157:H7. Subsequently, the cell culture medium from BMDMs and lysed BMDMs were used for agarose plating to assess bacterial proliferation. Figure 3E and F demonstrate that platelet co-incubation promoted the bacteria phagocytosis function of macrophages, resulting in reduced bacterial clones in the cell culture medium but more in lysed BMDMs agarose plating. Surprisingly, inhibition of EGFR activation using PD-168,393 or Erlotinib in platelets partially diminished the effect of platelets in promoting the bacteria phagocytosis function of macrophages. Similar observations were made with electron microscope analysis of BMDM macrophages. When co-incubated with platelets, BMDMs appeared more active in bacterial phagocytosis, exhibiting increased granules, lysosomes, and digestive vesicles. However, pre-inhibition of EGFR in co-incubated platelets appeared to fail to augment this immune function of macrophages (Fig. 3G). These data are consistent with the possibility that EGFR serves as an important mediator in platelets to regulate macrophage immune function at the site of inflammation and infection.

### EGFR of platelet promotes macrophage inflammation activation

**Fig. 4:**
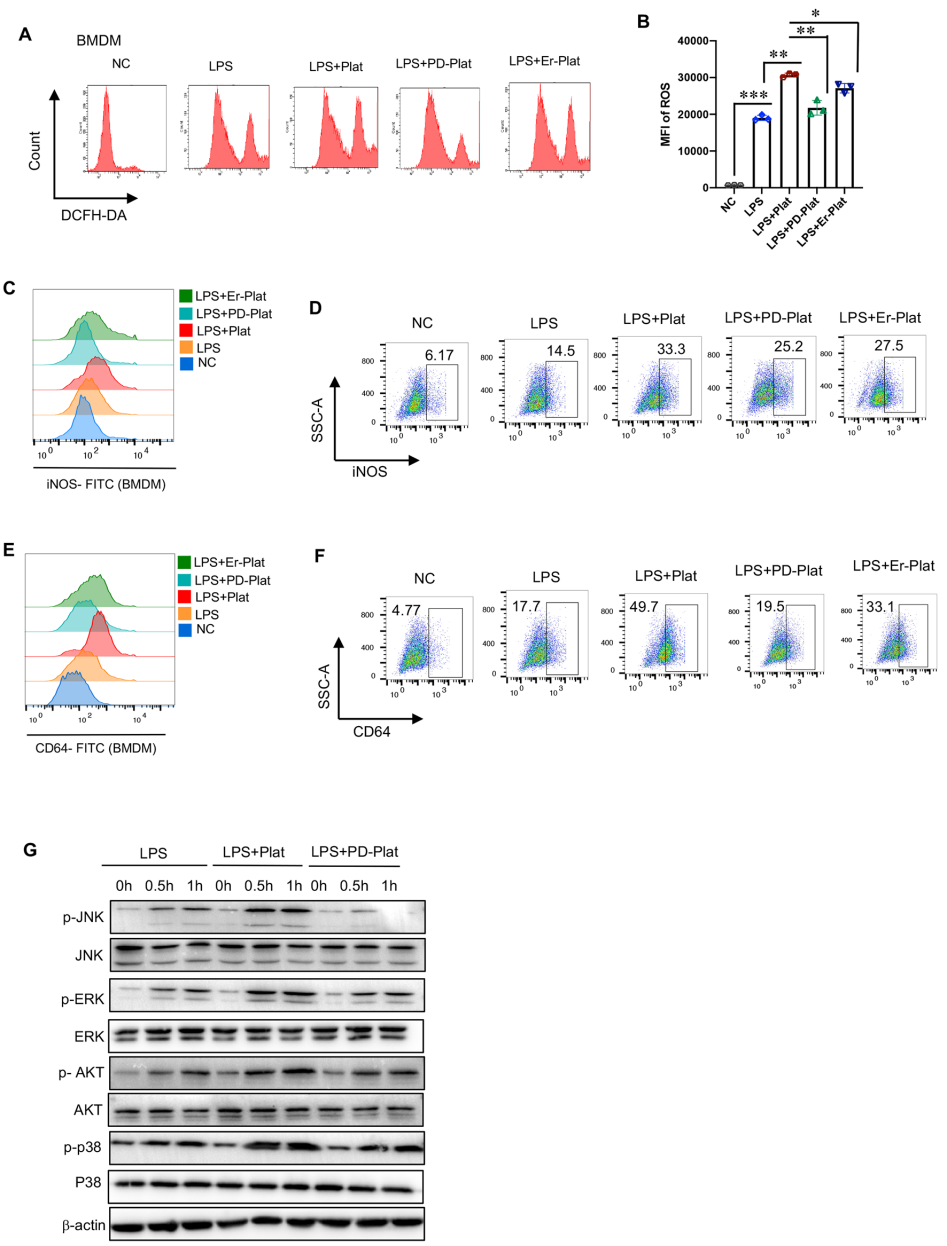
EGFR of platelet promotes macrophage inflammation activation. (**A-B**) ROS production of BMDM co-incubated with platelets upon LPS stimulation;. (**C-D**) iNOS expression of BMDM co-incubated with platelets upon LPS stimulation;. (**E-F**) CD64 expression of BMDM co-incubated with platelets upon LPS stimulation;. (**G**) Activation of the inflammation signaling pathway in BMDM co-incubated with platelets upon LPS stimulation was analyzed by Western Blotting of AKT, JNK, p38, ERK. (D: two tailed t-test * represents *P* < 0.05, ** represents *P* < 0.01, *** represents *P* < 0.001.)

Pro-inflammatory (M1) macrophages, characterized by high expression of reactive oxygen species (ROS), CD64, and inducible nitric oxide synthase (iNOS), are responsible for pathogen phagocytosis and killing [27]. Therefore, we further examined the possibility that platelet EGFR may promote pro-inflammatory macrophage M1 polarization. Flow cytometric analysis revealed that platelets increased ROS production in BMDMs upon LPS treatment, and EGFR inhibition with PD or Erlotinib on platelets was less effective in inducing ROS production in BMDMs (Fig. 4A-B). Similar findings were obtained for membrane iNOS and CD64 expression in BMDMs upon LPS treatment (Fig. 4C-F). Upon LPS stimulation, the phosphorylation of AKT, p38, c-Jun N-terminal kinase (JNK), and extracellular signal-regulated kinase (ERK) in BMDMs was considered indicative of activation of the inflammation pathway. Figure 4G demonstrated increased activation of these inflammation pathways in BMDMs after co-incubation with platelets upon LPS stimulation, and the EGFR inhibitor PD decreased the capacity of platelets to induce macrophage M1 polarization.

Taken together, these data strongly suggest that platelet EGFR likely plays a positive role in platelet-mediated macrophage M1 activation.

### EGFR of platelet regulates macrophage apoptosis and pyroptosis

Macrophages are capable of phagocytosing dying cells and cell debris, immune complexes, bacteria, and other waste products. However, they can also undergo cell death through various mechanisms in the complex environment associated with immune disorders such as sepsis [23, 28].

**Fig. 5:**
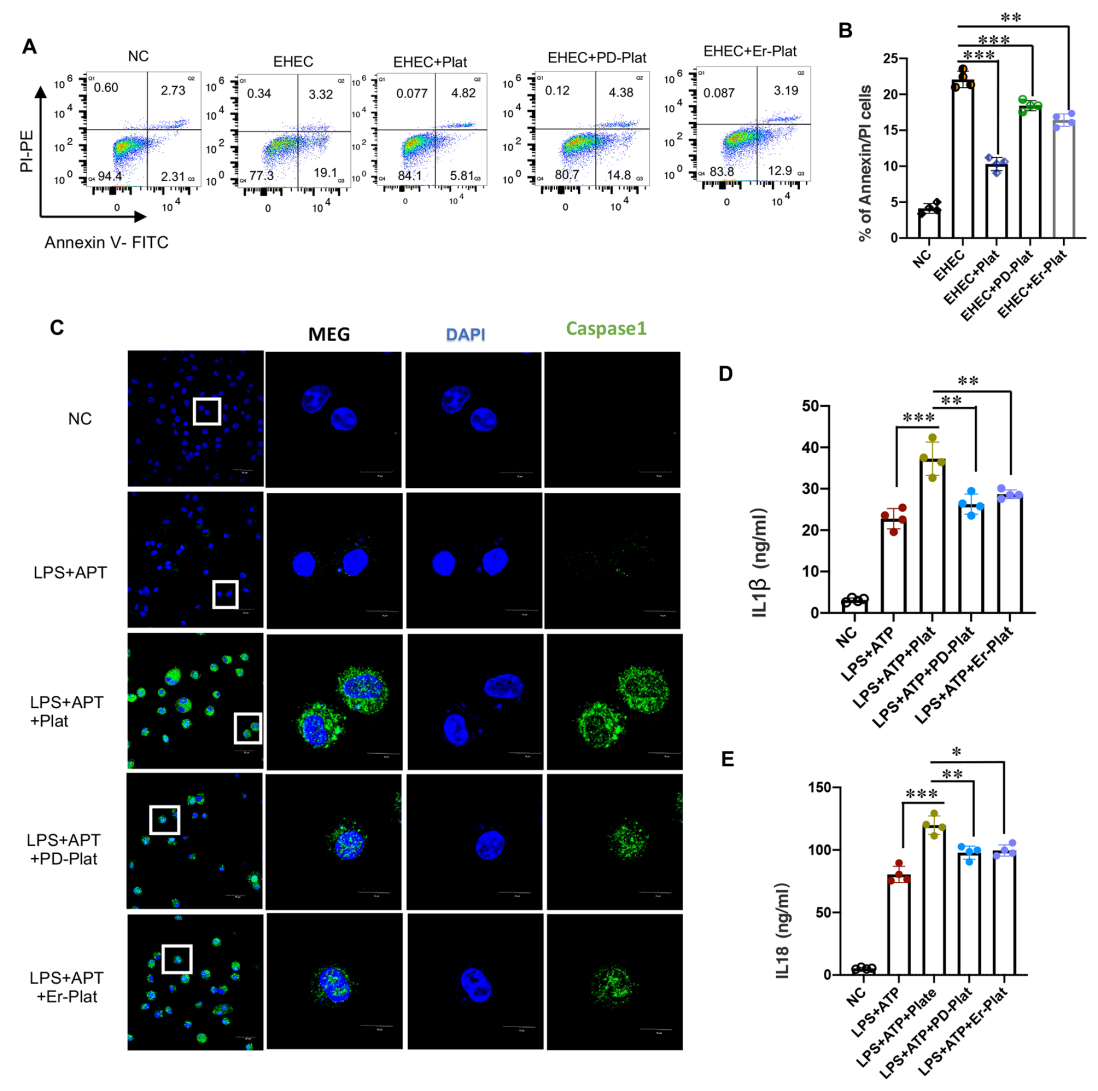
EGFR of platelet regulates macrophage apoptosis and pyroptosis. (**A-B**) Apoptosis population of BMDM with double Annexin V/PI staining with indicated treatments;. (**C**) Visualization of caspase1 in BMDM with indicated treatments. The images were acquired using Olympus FV3000 confocal microscope;. (**D-E**) IL-1β, IL18 secretion by BMDM with indicated treatments were measured by ELISA;. (B, D, E: two tailed t-test * represents *P* < 0.05, ** represents *P* < 0.01, *** represents *P* < 0.001.)

To analyze the impact of platelet EGFR on macrophage death mechanisms, we evaluated the role of EHEC-induced apoptosis and LPS + ATP-induced pyroptosis in BMDMs. In vitro experiments indicated that co-culture with platelets reduced the apoptosis of BMDMs, as evidenced by a decrease in the Annexin V/PI double-positive population and Annexin V-positive population upon EHEC infection. Moreover, inhibition of platelet EGFR by PD or Erlotinib partially reversed this phenotype (Fig. 5A-B). Interestingly, unlike apoptosis, co-culture with platelets increased the pyroptosis of BMDMs, as evidenced by the expression of caspase1 (Fig. 5C). Since IL-1β and IL-18 are produced during pyroptosis in macrophages, we further tested the production of these cytokines in BMDMs. Consistent with Fig. 5C, co-culture with platelets induced increased secretion of IL-1β and IL-18 in pyroptotic BMDMs, and inhibition of platelet EGFR by PD or Erlotinib partially reversed these phenomena (Fig. 5D-E).

These results underscore the intricate role of EGFR in platelets in determining the fate of macrophage immune functions and cell death mechanisms in sepsis.

## Discussion

When faced with pathogen invasion, macrophages must be capable of responding rapidly and phagocytizing bacteria to protect the host [27, 29]. The critical process requires efficient immune activation of M1 macrophages, which is regulated by complex mechanisms [9].

In this study, we have demonstrated that platelets regulate the inflammatory macrophage response by promoting macrophage bacterial clearance function and reprogramming macrophages toward the M1 phenotype through EGFR. Moreover, EGFR also plays important role in platelet activation by increasing CD62L expression. Importantly, platelet overexpression by TPO treatment educedrs mortality in CLP-induced sepsis model.

Platelets decrease the production of TNF-a by murine bone marrow-derived macrophages when exposed to high concentrations of LPS. Additionally, platelets prompt an anti-inflammatory reaction in murine macrophages [13]. The presence of activated platelets has been observed to exacerbate pro-inflammatory macrophage activation. The interaction between macrophages and autologous platelets enhances LPS-induced cytokines of macrophage [14]. Here, we demonstrate that platelet overexpression induced by TPO treatment leads to a reduction in both pro- and anti-inflammatory responses in the lung, kidney, liver, and spleen in a sepsis model. This phenomenon may be attributed to the ability of platelets to sequester monocyte-derived cytokines.

Interestingly, we further observed that platelets positively regulate the macrophage bacterial clearance function mediated by platelets and promote M1 polarization.

Notably, platelets promote the invasion of monocytes-macrophages by B.abortus infection. Platelets exhibited the ability to reprogram monocytes towards pro-inflammatory phenotype through a cell-contact-dependent mechanism [30]. Additionally, platelets have been observed to encapsulate bacteria via a mechanism involving a ‘touch-and-go’ interaction between GPIb and von Willebrand factor expressed on Kupffer cells in the liver sinusoids [31]. The suggested platelet surveillance mechanism is believed to play a role in swift host defense and early survival against specific blood pathogens.

Our data highlight a new mechanism through which platelets may contribute to the rapid bacterial clearance by macrophages. It has been demonstrated that EGFR regulates the inflammatory function of macrophages, and EGFR can also induce the apoptosis of CD4(+) T lymphocytes through the TBK1/Glut1-induced Warburg effect in sepsis [19, 20, 32]. Here, our data indicate that EGFR is necessary not only for the activation of platelets themselves but also to some extent for triggering platelet-mediated bacteria phagocytosis and M1 macrophage polarization. We have identified that the beneficial effect of EGFR in platelets is attributed to an increase in iNOS + M1 macrophages, which contribute to efficient bacterial clearance. Importantly, these discrepancies could be associated with sepsis mortality.

Macrophages have the capability to phagocytose dying cells, cell debris, immune complexes, bacteria, and other waste products. However, in the complex immune-disordered environment of sepsis, they are susceptible to various forms of cell death. Therefore, we speculated that EGFR on platelets may regulate the manner of macrophage death, enabling a bidirectional regulation that could help elucidate the complex mechanisms of sepsis, which are still poorly understood. Interestingly, we found that EGFR on platelets reduced the apoptosis of bone marrow-derived macrophages (BMDM) induced by EHEC infection but increased pyroptosis of macrophages upon treatment with LPS + ATP. However, since these experiments were only conducted in vitro using EGFR inhibitors, further studies are necessary to gain a deeper understanding of the EGFR-mediated pathway during platelet-induced macrophage death.

**Fig. 6:**
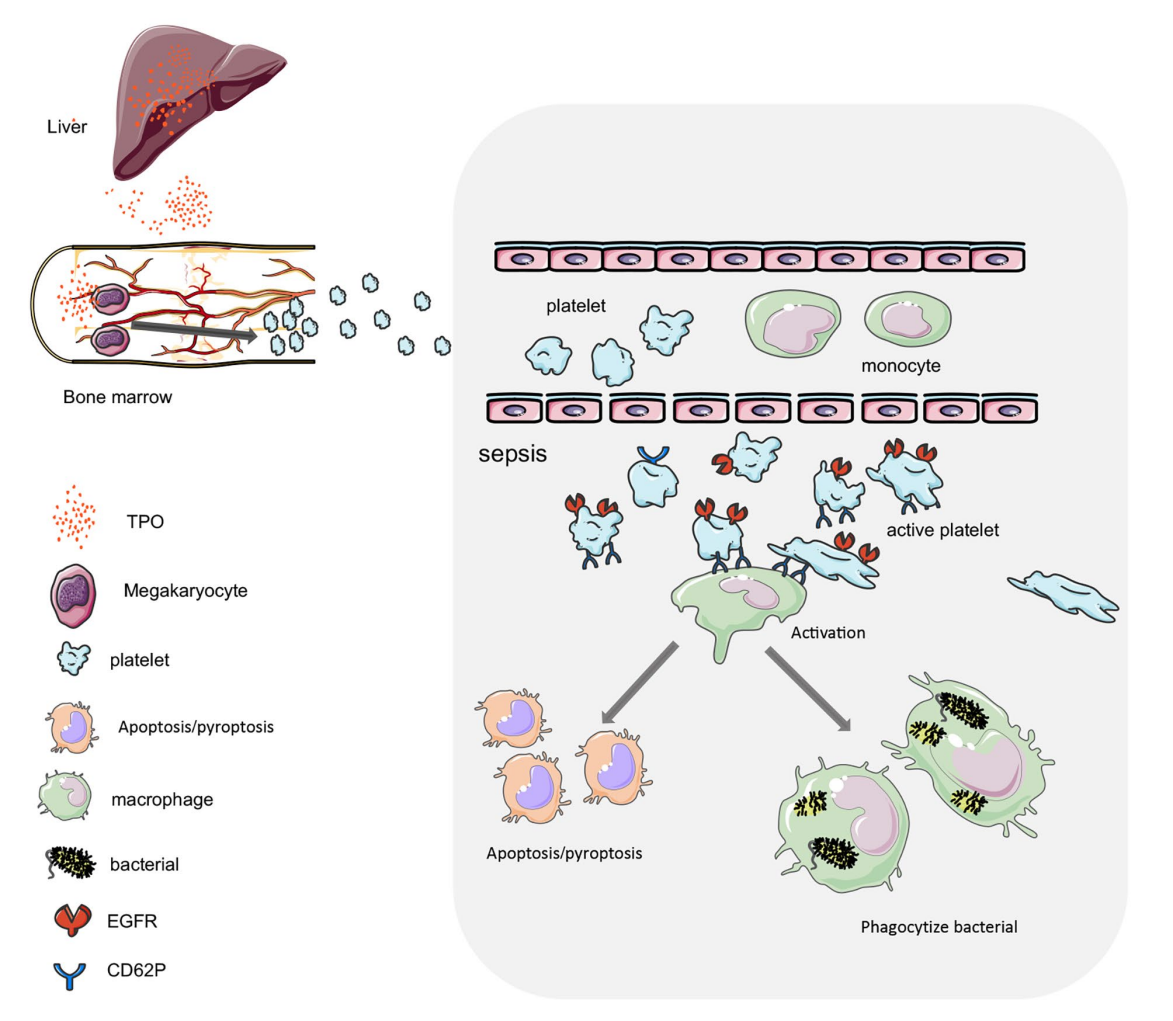
Working model for EGFR of platelet regulated macrophage activation

In addition to their well-established roles in hemostasis and thrombosis, platelets are now recognized as vital contributors during infection. They play crucial roles in disrupting tissue integrity and contributing to inflammation, pathogen elimination, and tissue repair [33, 34]. This advancement has offered a new perspective for our understanding of sepsis pathophysiology. Therefore, the role of platelets extends beyond direct interactions with pathogens. Our findings highlight the central role of EGFR in platelet-macrophage interactions, which is crucial for reprogramming macrophage immune function and fostering antimicrobial activity during sepsis. (Fig. 6). These findings may have implications for EGFR-targeted treatments aimed at platelets and immune cells in sepsis, as well as in a wide range of pathophysiological conditions, including chronic inflammation, cancer, thrombosis, and vascular-related diseases.

## Materials and methods

### Mice

C57BL/6 (WT) mice were obtained from Jiangsu Jicui Yaokang Biotechnology Co., Ltd (Jiangsu, China). Mice were housed according to protocols approved by the Animal Ethics Committee of Guangdong Medical University.

### Treatments

The cecal ligation and puncture (CLP) mouse model operative procedures were conducted following previously established methods (Marco et al., 2018). In brief, C57BL/6 female mice were anesthetized with general isoflurane (2%) anesthesia. The cecum was perforated, leading to the release of fecal material into the peritoneal cavity.

The PT3-F1α-TPO lentiviral plasmid was generously provided by Dr. Zhanghui Chen (Institute of Clinical Medicine, Zhanjiang Central Hospital, Guangdong Medical University, Zhanjiang, China). To induce TPO overexpression in mice, a hypertensive tail vein injection of 10 μl PT3-EF1α-TPO lentiviral plasmid with a titer of 1 × 10^11 virus was administered.

### Preparation of mouse platelets

Blood was collected from the abdominal aortas of mice under isoflurane anesthesia using anticoagulant tubes. Platelet isolation was carried out using the Mouse Peripheral Blood Platelet Separation Kit (PLA2011M, Haoyang, Tianjin, China) following the manufacturer’s instructions. Platelet counts in whole blood were determined using a HEMAVET HV950FS multispecies hematology analyzer.

### Mouse BMDM and cell treatment

BMDMs were isolated from the femurs and tibias of C57BL/6 mice, following previously established protocols (ref 19). The cells were cultured in DMEM supplemented with 10% FBS and 1% (v/v) penicillin/streptomycin.

For co-culture experiments with platelets, washed platelets at a concentration of 1 × 10^9 were added to the BMDM culture medium and incubated for 2 h, with or without pretreatment of PD-16,839 (10 μM) or Erlotinib (20 μM).

To induce pyroptosis in BMDMs in vitro, LPS (1 μg/ml) and ATP (4 mM) were added to the BMDM culture medium and incubated for 4 h.

For in vitro infection with E. coli, E. coli O157:H7 (provided by Dr. Tianwen, Guangdong Medical University, Zhanjiang, China) was cultured in Luria-Bertani (LB) medium. Prior to infection, cells were washed extensively with 1X PBS and then incubated in complete DMEM without antibiotics for 2–3 h. The concentration of the bacterial solution was determined using a standardized calibration curve of OD 600/colony-forming units (CFU).

### Flow cytometry analyses

Cell surface staining and flow cytometry were performed as described previously (ref 19).

Flow cytometry acquisition was conducted using a FACS cytometer (BD Biosciences, USA), and data processing was carried out using FlowJo version 10.0 software.

### Western blotting

Following the indicated treatments, cell supernatants were collected and lysed in RIPA lysis buffer (Beyotime, China) supplemented with Protease Inhibitor Cocktail and Phosphatase Inhibitor Cocktail. Protein concentration was quantified using the BCA protein assay (Beyotime, China).

### Quantitative real-time PCR

Total RNA was extracted from 5 × 10^5 cells following the indicated treatments using TRIzol (Invitrogen), according to the manufacturer’s instructions. cDNA was synthesized from each RNA sample using the TaKaRa reverse transcriptase kit. Quantitative PCR (qPCR) was carried out using TB Green Premix Ex Taq II (TaKaRa) on a Roche 480 instrument. Primers used are listed in Supplemental Table 1.

### ELISA

Cell supernatants, mice plasma, or mice organs were collected after the indicated treatments. The concentrations of cytokines, including mouse IL-6, mouse TNF-α, mouse IL-18 and mouse IL-1β, were measured using ELISA kits (Thermo Scientific) following the manufacturer’s instructions. All ELISA assays were conducted according to the manufacturer’s protocols.

### Immunofluorescence staining

Bone marrow murine neutrophils (6 × 10^5) were plated on confocal dishes. Following the indicated stimulation, cells underwent immunofluorescence staining procedures. Confocal microscopy was performed using an Olympus FV3000 confocal microscope.

### Transmission electron microscopy

Mice subjected to the indicated treatments were euthanized and immediately perfused with normal saline followed by 4% paraformaldehyde solution. The lung, liver, and spleen were fixed in 2% glutaraldehyde at 4 °C overnight. Subsequently, target tissues were dissected, and selected areas were further fixed in 1% osmium tetroxide for 1 h. Following fixation, samples were dehydrated in a graded ethanol series, embedded in epoxy resin, and polymerized at 80 °C for 24 h. Ultrathin Sect. (100 nm) were cut using a Leica EM UC7 and placed on EM grids. Sections were stained with uranyl acetate and lead citrate before examination with an EM-1400 transmission electron microscope (Japan).

### Colony formation experiment

E. coli was cultured at 37 °C under aerobic conditions with shaking in Luria Bertani (LB) medium overnight. The bacteria were added to macrophage cultures at a ratio of 1:2500 (v/v) when the bacterial culture reached an OD of 0.6 as determined by spectrophotometry, followed by incubation for 1 h at 37 °C. Subsequently, cell medium or cell lysates were collected for the colony formation experiment. Image capture was conducted using an EVOS XL Core Microscope (Life Technologies).

LB medium was prepared by dissolving 1 g tryptone, 0.5 g yeast extract, 1 g NaCl, and 1.4 g agar powder in 100 ml distilled water, followed by sterilization at 121 °C for 25 min. LB medium was then poured into petri dishes in an ultra-clean workbench and allowed to cool and solidify.

Equal volumes of BMDM medium or cell lysates from peritoneal lavage fluid of each group were added onto the solid LB medium and spread evenly with a sterile spreader until dry. The amount of liquid added to the plate was adjusted according to the number of colonies in the culture medium. The coated LB plates were sealed with sealant and incubated at 37 °C overnight for 24 h to observe the number of colonies.

### Histological analysis

Tissues were formalin-fixed, processed, and paraffin-embedded. H&E staining was performed by Hangzhou Luoke Biotechnology Co., Ltd.

### Statistical analysis

Statistical analyses were performed using Prism software (GraphPad 9), and *P* values were calculated using a two-tailed t-test or one-way ANOVA analysis, as appropriate. Each experiment was independently repeated at least three times, and the data are presented as the mean ± standard error (SE). Statistical significance is denoted by asterisks (*P < 0.05; **P < 0.01; ***P < 0.001).

### Electronic supplementary material

Below is the link to the electronic supplementary material.

**Supplementary Material 1:** Key Resources table

**Supplementary Material 2:** Original full-length gel and blot images

**Supplementary Material 3:** Figure. S1. TPO-elevated platelets increased platelet expression in C56BL/6 mice

## Data Availability

All data generated or analyzed during this study are included in this published article (and its supplementary information files).

## Abbreviations

BMDM: Bone Marrow Derived Macrophage
CLP: Cecal ligation and puncture
EGFR: Epidermal growth factor receptor
EHEC: E. coli O157:H7
ERK: Extracellular signal–regulated kinase
IL1β: Interleukin1β
IL18: Interleukin18
IL6: Interleukin 6
iNOS: Inducible nitric oxide synthase
JNK: C-Jun N-terminal kinase
LPS: Lipopolysaccharide
PD: PD-168393
ROS: Reactive oxygen species
TLR4: Toll-like receptor 4
TPO: Thrombopoietin
