# Supplementary material for: EGFR of platelet regulates macrophage activation and bacterial phagocytosis function
Source: J Inflamm (Lond). 2024 Apr 17;21:10. doi: 10.1186/s12950-024-00382-1 (PMC11022435; doi:10.1186/s12950-024-00382-1)
Supplement: Supplementary file 1 — Supplementary Material 1: Key Resources table [file 12950_2024_382_MOESM1_ESM.docx]

**KEY RESOURCES TABLE**

**Key resources table**

| REAGENT or RESOURCE | SOURCE | | IDENTIFIER |
| --- | --- | --- | --- |
| Antibodies | | | |
| Thrombopoietin（TPO） Polyclonal Antibody | Thermo Fisher | | **PA5-80125** |
| anti-EGFR-Antibody | abcam | | ab52894 |
| β-Actin (8H10D10) Mouse mAb #3700 | CST | | #3700 |
| TLR4 Monoclonal Antibody | Santa Cruz Biotechnology | | 52962 |
| Caspase 1 Monoclonal Antibody (B0-6) | Thermo Fisher | | MA5-32909 |
| NF-κB p65 Monoclonal antibody | proteintech | | Cat No. 66535-1-Ig |
| Anti-NF-kB p65 antibody [E379] (ab32536) | abcam | | ab32536 |
| Anti-NF-kB p65 (phospho S536) antibody [EP2294Y] (ab76302) | abcam | | ab76302 |
| Phospho-SAPK/JNK (Thr183/Tyr185) (81E11) Rabbit mAb #4668 | CST | | 4668T |
| β-Actin (8H10D10) Mouse mAb #3700 | CST | | #3700 |
| Phospho-p38 MAPK (Thr180/Tyr182) (D3F9) XP® Rabbit mAb #4511 | cst | | #4511 |
| PE Goat anti-mouse Ig G | Biolegend | | 405307 |
| FITC anti-mouse/rat CD62P (P-selectin) Antibody | Biolegend | | 148303 |
| CD11B-FITC | BD Pharmingen | | 557396 |
| PerCP/Cyanine5.5 anti-mouse CD45 Antibody | Biolegend | | 103132 |
| PE anti-mouse F4/80 Recombinant Antibody | Biolegend | | \| 157303 \| \| --- \|   157303 |
| FITC anti-Nos2 (iNOS) Antibody | Biolegend | | 696805 |
| FITC anti-mouse CD64 Antibody | Biolegend | | 161007 |
|  |  | |  |
| Bacterial and virus strains | | | |
| E. coli O157:H7 | Ph.D. Tianwen Lai, Department of Respiratory and Critical Care Medicine, Affiliated hospital of Guangdong Medical University, Zhanjiang, Guangdong, China. | |  |
|  |  | |  |
|  |  | |  |
| Chemicals, peptides, and recombinant proteins | | | |
| PD-168393 | Millipore | | 513033 |
| Erlotinib | Selleck | | S1023 |
| Lipopolysaccharide | Sigma-Aldrich | | L2880 |
|  |  | |  |
| Critical commercial assays | | | |
| Mouse Peripheral Blood Platelet Separation Kit | Haoyang,Tianjin，China | | PLA2011M |
| Mouse TNF alpha Uncoated ELISA | Thermo Scientific | | 88-7324 |
| Mouse IL-6 Uncoated ELISA | Thermo Scientific | | 88-7064 |
| Mouse IL-1 beta Uncoated ELISA | Thermo Scientific | | 88-7013 |
| Reactive oxygen species assay kit | Bioss | | BC01010 |
|  |  | |  |
| Experimental models: Cell lines | | | |
|  |  | |  |
|  |  | |  |
|  |  | |  |
|  |  | |  |
|  |  | |  |
| Experimental models: Organisms/strains | | | |
| C57BL/6 mice | GemPharmatech^M^, China | |  |
|  |  |  |  |
|  |  | |  |
| Oligonucleotides | | | |
| qPCR primers for indicated genes | This paper | |  |
| m-β‐actin-F | CCTTCTTGGGTATGGAATCCTGT | |  |
| m-β‐actin-R | GGCATAGAGGTCTTTACGGATGT | |  |
| m-IL-10-F | GGTTGCCAAGCCTTATCGGA | |  |
| m-IL-10-R | GGGGAGAAATCGATGACAGC | |  |
| m-IL-1b-F | TGCCACCTTTTGACAGTGATG | |  |
| m-IL-1b-R | TGATGTGCTGCTGCGAGATT | |  |
| m-TNFα-F | AAGCCTGTAGCC CACGTCGTA | |  |
| m-TNFα-R | GGCACCACTAGTTGGTTGTCTTTG | |  |
| m-IL-6-F | TAGTCCTTCCTACCCCAATTTCC | |  |
| m-IL-6-R | TTGGTCCTTAGCCACTCCTTC | |  |
|  |  | |  |
| Recombinant DNA | | | |
| PT3-EF1α-TPO lentiviral plasmid | Ph.D. Zhanghui Chen, Institute of Clinical Medicine, Zhanjiang Central Hospital, Guangdong Medical University, Zhanjiang, China. | |  |
|  |  | |  |
| Software and algorithms | | | |
| ﻿GraphPad Prism 9 | ﻿GraphPad Software | | ﻿http://www.bitplane.com/imaris/imaris |
| ﻿FlowJo 10 | ﻿TreeStar | | ﻿http://flowjo.com/ |
| Adobe Photoshop CC | Adobe Photoshop | | <http://ps.chenqix.cn/?bd_vid=8420984592824528360> |
|  |  | |  |
| Other | | | |
|  |  | |  |
|  |  | |  |
|  |  | |  |
|  |  | |  |
|  |  | |  |
