# Supplementary figures and images for: EGFR of platelet regulates macrophage activation and bacterial phagocytosis function
Source: J Inflamm (Lond). 2024 Apr 17;21:10. doi: 10.1186/s12950-024-00382-1 (PMC11022435; doi:10.1186/s12950-024-00382-1)

Figure 1B

NC PV P-THPO

NC PV P-THPO

NC PV P-THPO


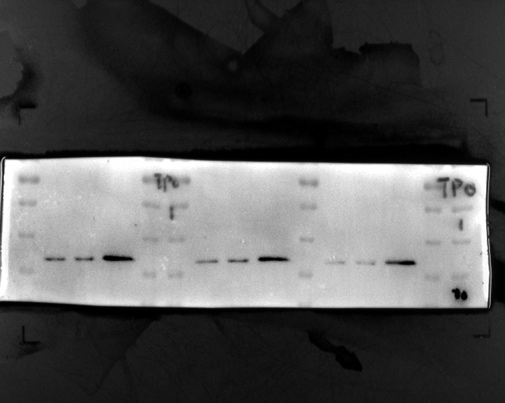


TPO


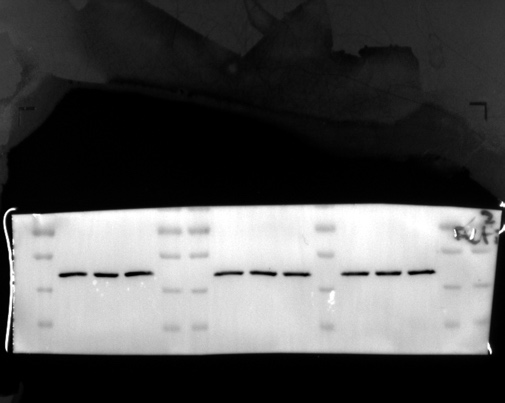


Actin

Figure 4G


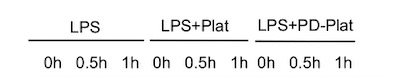


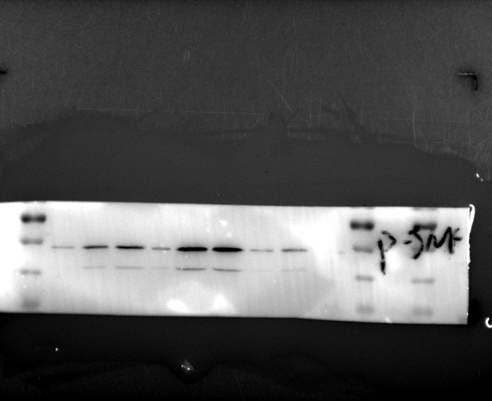


p-JNK


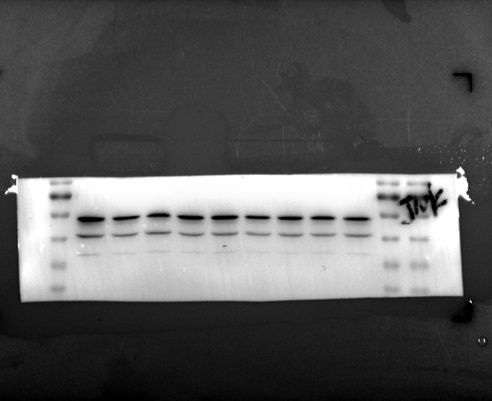


JNK


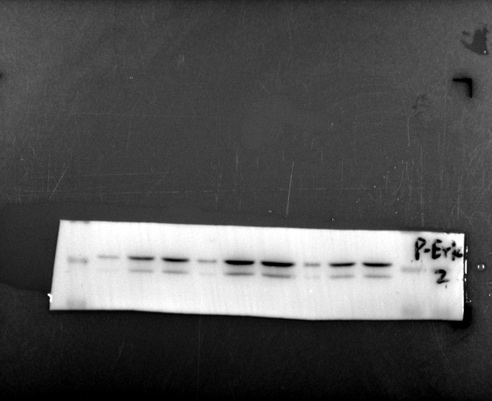


p-ERK


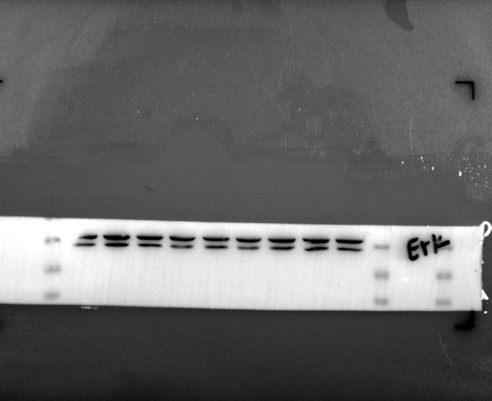


ERK


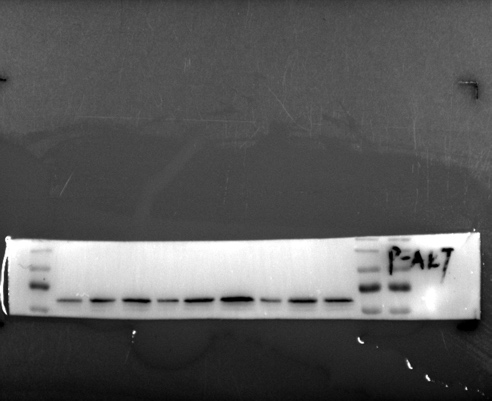


p-AKT


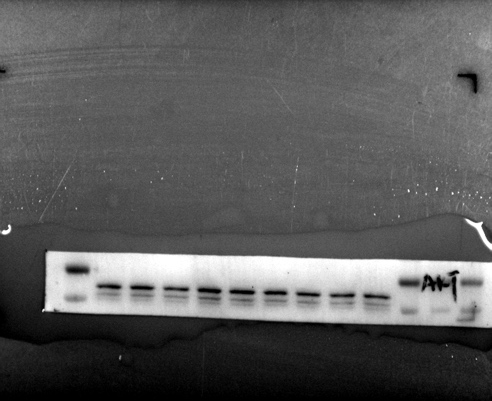


AKT


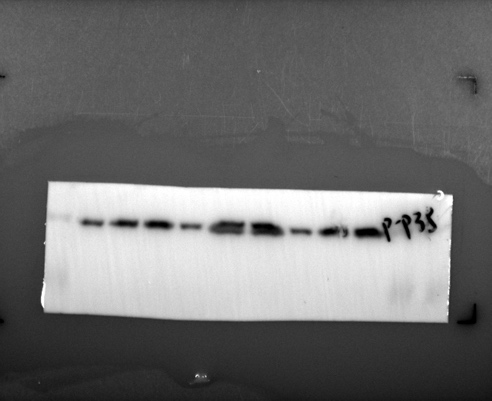


p-p38


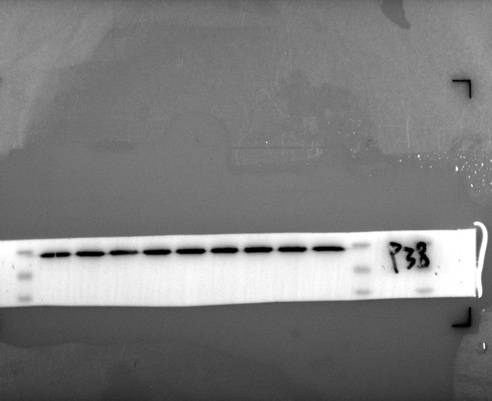


p38


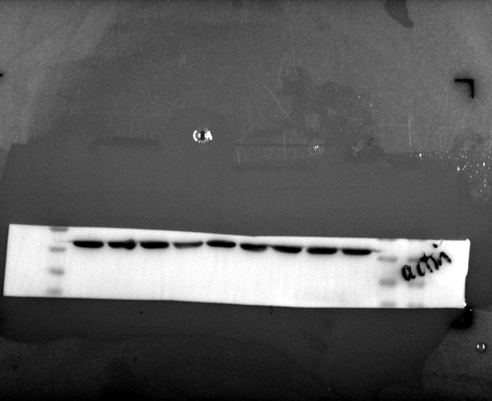


Actin

Supplement: Supplementary file 2 — Supplementary Material 2: Original full-length gel and blot images [file 12950_2024_382_MOESM2_ESM.docx]
