# Supplementary material for: EGFR of platelet regulates macrophage activation and bacterial phagocytosis function
Source: J Inflamm (Lond). 2024 Apr 17;21:10. doi: 10.1186/s12950-024-00382-1 (PMC11022435; doi:10.1186/s12950-024-00382-1)
Supplement: Supplementary file 3 — Supplementary Material 3: Figure. S1. TPO-elevated platelets increased platelet expression in C56BL/6 mice [file 12950_2024_382_MOESM3_ESM.docx]

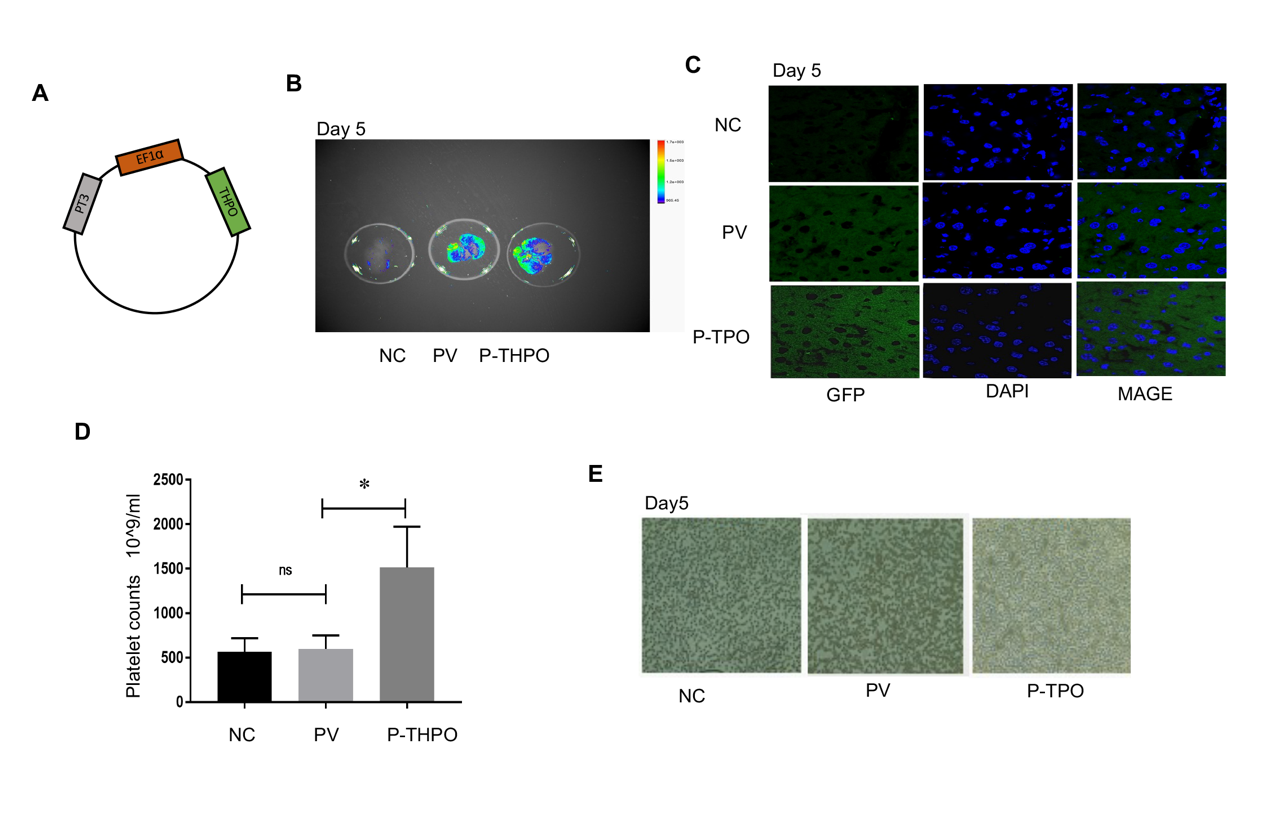


**Figure. S**1. TPO-elevated platelets increased platelet expression in C56BL/6 mice.

**(A)** Simple schematic diagram of e. coli clone plasmid of PT3-EF1α-TPO.

**(B)** General GFP green fluorescence distribution of fresh isolated mouse livers detected 5 days after TPO-plasmid tail vein injection;

**(C)** The distribution of GFP green fluorescence in mouse hepatocytes was detected by confocal imaging system 5 days after TPO-plasmid injection;

**(D)** The number of platelets in peripheral blood of mice;

**(E)** Platelets increased in peripheral blood smears 5 days after TPO-plasmid injection.

(D: two tailed t-test * represents P<0.05, ** represents P<0.01, *** represents P<0.001.)
